# Single-cell RNA-sequencing of virus-specific cellular immune responses in chronic hepatitis B patients

**DOI:** 10.1038/s41597-024-03187-2

**Published:** 2024-04-08

**Authors:** Klas Hatje, Tony Kam-Thong, Nicolas Giroud, Antonio Saviano, Pauline Simo-Noumbissie, Nadine Kumpesa, Tobias Nilsson, François Habersetzer, Thomas F. Baumert, Nadege Pelletier, Marianne Forkel

**Affiliations:** 1 Roche Pharma Research and Early Development, Pharmaceutical Sciences, Roche Innovation Center Basel, Basel, Switzerland; 2 Service d’hépato-gastroentérologie, Pôle hépato-digestif, Institut Hospitalo-Universitaire de Strasbourg, Hôpitaux Universitaires de Strasbourg, Strasbourg, France; 3 Institut de Recherche sur les Maladies Virales et Hépatiques, Inserm UMR_S1110, University of Strasbourg, Strasbourg, France; 4 Roche Pharma Research and Early Development, Immunology, Infectious Diseases and Ophthalmology (I2O) Discovery and Translational Area, Roche Innovation Center Basel, Basel, Switzerland

**Keywords:** Hepatitis B, Transcriptomics, T cells, Data integration

## Abstract

Chronic hepatitis B (CHB) is a major global health challenge. CHB can be controlled by antivirals but a therapeutic cure is lacking. CHB is characterized by limited HBV-specific T cell reactivity and functionality and expression of inhibitory receptors. The mechanisms driving these T cell phenotypes are only partially understood. Here, we created a single-cell RNA-sequencing dataset of HBV immune responses in patients to contribute to a better understanding of the dysregulated immunity. Blood samples of a well-defined cohort of 21 CHB and 10 healthy controls, including a subset of 5 matched liver biopsies, were collected. scRNA-seq data of total immune cells (55,825) plus sorted HBV-specific (1,963), non-naive (32,773) and PD1^+^ T cells (96,631) was generated using the 10X Genomics platform (186,123 cells) or the full-length Smart-seq2 protocol (1,069 cells). The shared transcript count matrices of single-cells serve as a valuable resource describing transcriptional changes underlying dysfunctional HBV-related T cell responses in blood and liver tissue and offers the opportunity to identify targets or biomarkers for HBV-related immune exhaustion.

## Background & Summary

### Background

Hepatitis B virus (HBV) infection is a major global public health problem despite the existence of safe and effective preventative vaccines. More than 250 million people worldwide are living with chronic HBV. Patients carry a high risk of cirrhosis and hepatocellular carcinoma (HCC)^1^. Current treatments are based on direct antiviral agents which can only limit HBV replication without achieving a long-term HBV cure^2^. Ineffective immune responses are a key feature enabling chronic HBV infection but also contributing to hepatocytes injury and liver inflammation. Understanding the mechanisms behind this dysfunctional immune response and the switches towards a functional response resulting in viral cure will be key for the development of new HBV treatments such as immunotherapies.

During acute infection a robust immune response involving HBV-specific antiviral CD8^+^ effector cells, CD4^+^ helper T cells and B cells producing HBV-specific antibodies leads to viral clearance and resolution of infection^3–6^.

However, during chronic HBV infection, immune responses show a number of dysregulated features including atypical B cells producing reduced antibody levels and T cell exhaustion, characterized by low levels of HBV-specific T cells with a state of low functionality^7–10^. T cell exhaustion is defined by restricted proliferation, progressively reduced cytotoxicity and cytokine production and increased expression of inhibitory receptors^9,11–14^.

Chronic exposure to high antigen levels, extended duration of disease as well as suboptimal T cell priming and activation have been identified as some of the factors driving this dysfunctional immune response^14–18^.

Recently, it has become clear that exhausted T cells in chronic HBV infection are not representing a homogeneous cell population^19–21^.

Data from a small cohort of patients achieving HBV functional cure either spontaneously or after cessation of NUC treatment shows that induction of a sufficient immune response is possible even in a setting of chronic HBV infection^22,23^.

Many studies on immune responses in HBV patients have focused on the analysis of peripheral blood immune cells due to easier sample accessibility^9,11,13,16^. However, HBV is a purely hepatotropic virus and it has become clear that the cell composition and functional features of intrahepatic immune cells can differ substantially from the ones in peripheral blood^24–28^.

Single-cell RNA-sequencing (scRNA-seq) is a breakthrough technology allowing for transcriptome analysis cell by cell. This technique has been widely used to assess immune cell profiles in chronic infectious diseases or cancers related to an immune exhaustion phenotype^29–32^. ScRNA-seq, as an unbiased transcriptome analysis, provides also a great opportunity to discover the underlying cellular and molecular mechanisms contributing to the dysfunctional immune response in chronic HBV infection.

So far, only few studies have explored the HBV immune landscape with scRNA-seq in human samples. Zheng *et al*. explored sorted T cells infiltrating HCC tumor tissue on an HBV background^33^. Zhang and colleagues focused on the liver immune cell infiltration during different phases of chronic HBV infection in an Asian patient cohort^34^. A pilot study from Genshaft *et al*. explored the technological feasibility of using fine needle aspirates for scRNA-seq comparing two different methods^35^ and the latest sc-RNAseq study in the HBV field investigated changes in the immune cell landscape during the progression from HBV infection to HBV cirrhosis and HBV-associated HCC^36^.

**Fig. 1:**
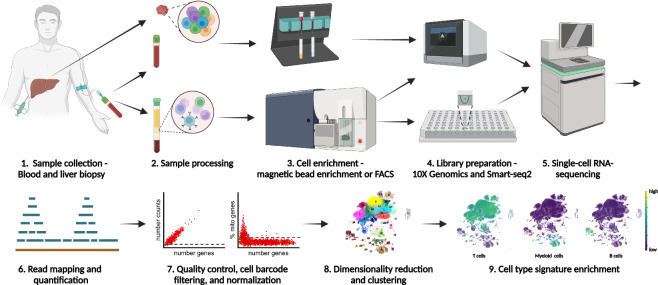
Schematic overview of the study workflow. 1. Blood samples from patients with chronic hepatitis B infection were collected. Matched liver biopsies were available for a subgroup of patients. Blood from healthy donors was used as control. 2. For liver biopsies, single cell suspensions were prepared by enzymatic digestion. PBMCs were isolated by density centrifugation. A subsample of whole blood was used in parallel with the liver cell suspension for magnetic bead isolation. 3. CD45^+^ cells were isolated using magnetic bead enrichment from both whole blood and liver cell suspensions. Specific T cell populations (Non-naive, PD1^+^ or HBV-specific T cells) were sorted from PBMCs via FACS sorting either in plates as single cells per well or as bulk populations in tubes. 4. Libraries for 3′ scRNA-seq were prepared from CD45^+^ cells and sorted T cell populations using the 10X Genomics Chromium platform. Libraries for full-length RNA-sequencing using the Smart-seq2 protocol were prepared from plate-sorted T cells. 5. scRNA-seq was performed using the Illumina NovaSeq instrument. 6. Sequencing reads were mapped to the human genome and a gene-by-cell count matrix was generated. 7. Sample quality was assessed and cell barcodes were filtered based on number of genes, read or UMI counts, and mitochondrial content. The gene-by-cell count matrix was normalized to counts per 10’000 (cp10k). 8. Highly variable genes were selected, principal components analysis was performed, nearest neighbors were identified, Leiden clustering and uniform manifold approximation projection (UMAP) were performed. 9. Signature enrichment scores were calculated to identify cell types.

In this study, we established a scRNA-seq dataset of total immune cells and HBV-specific T cells from blood and liver of non-cirrhotic HBV patients without HCC (Fig. 1). We used both a droplet-based microfluidic scRNA-seq system (10X Genomics) and full-length scRNA-seq by Smart-seq2 to analyze total immune cells and HBV-specific T cells.

To the best of our knowledge, this dataset represents very comprehensive scRNA-seq data focusing on the complete immune compartments in blood and liver of non-cirrhotic HBV patients without HCC and is the only published dataset including full-length sequencing of sorted HBV-specific T cells.

As such our data is a valuable resource for the scientific community to better understand the mechanisms underlying the dysfunctional immune response during chronic HBV infection contributing to the development of new treatment strategies for this disease.

## Methods

### Human clinical samples

Samples from two different groups of patients were included in the study. From 15 NUC treated CHB patients (HBeAg negative, HBsAg positive, HBV DNA < 25 IU/mL, ALT < 1.5 × UNL) 100 mL of blood were taken. From 6 untreated CHB patients (HBeAg negative, HBsAg positive, HBV DNA > 2000 IU/mL, ALT < 2x UNL) 100 mL blood as well as one matched liver biopsy for 5 of them was obtained. All patients were treated and followed at the Strasbourg University Hospital, France. Patient characteristics are shown in Table 1.

**Table 1:** Characteristics of the study cohort.

Patient ID	Age	Sex	Biopsy	Fibrosis	ALT (IU/mL)	AST (IU/mL)	Total bilirubin (µmol/L)	INR	Albumin (g/L)	platelets (G/L)	Disease duration (years)	HBV genotype	HBsAg (IU/mL)	HBV DNA (IU/ml)	Antiviral treatment	Duration antiviral treatment (years)
1	65	F	no	F0-F1	27	19	NA	NA	NA	242	7	NA	351.31	<10	TDF	7
2	44	M	no	F0-F1	96	60	16	1	45	251	15	NA	23547.07	265210	No	NA
3	55	M	no	F0-F1	24	15	7.5	1	54	219	15	B	405.32	<10	ETV	13
4	26	M	no	F0-F1	32	23	18.7	1	54	203	25	NA	43715.09	<10	Yes, NA	6
6	39	M	no	F1-F2	26	26	12.5	NA	45	211	10	E	17581.84	<10	ETV	5
7	39	M	no	F0-F1	24	19	24.2	1.1	42	196	9	E	3448.37	<10	TDV	8
8	39	F	no	F0-F1	17	18	13.3	NA	44	285	18	NA	8836.84	<10	TDV	16
9	68	M	no	F0-F1	16	26	8.5	NA	45	251	23	NA	970	<10	TDV	19
10	40	F	no	F0-F2	29	41	NA	NA	NA	248	4	NA	24015.82	<10	TDV	1
11	35	M	no	F0-F1	19	19	7.2	1	40	ND	7	D	9717	<10	TDV	6
12	45	F	no	F0-F1	29	19	8.2	1	NA	384	24	NA	1980.26	<10	TDV	7
14	57	M	no	F2	82	47	NA	NA	NA	ND	6	NA	256	<11	TDV	7
16	39	F	no	F0-F1	29	29	2.2	1	46	255	11	D	20991	<10	TDV	7
21	44	F	yes	F0	31	26	10.2	1	45	261	27	NA	1608	899100	No	NA
26	21	M	yes	0	58	42	14	1.2	NA	219	13	E	2896.87	11457	No	NA
27	31	M	no	NA	33	31	10.2	NA	43	156	NA	NA	1540.41	<10	ETV	10
28	54	M	no	F0-F1	21	21	14.3	1.2	46	194	33	D	2122	<10	ETV	11
34	23	M	yes	F0	84	24	14.9	1	51	211	2	NA	1109.93	11162	No	NA
35	39	M	yes	F0	43	26	32.3	1	47	239	5	E	27138.56	40773	No	NA
36	69	M	no	F0-F1	19	27	13.9	1.03	44	266	36	NA	2605	<10	ETV	7
37	24	M	yes	F0	224	114	16	1	46	306	4	NA	22740.11	349793	No	NA

Abbreviations: ALT - alanine aminotransferase, ETV – entecavir, F - female, M - male, NA - not available, TDF – tenofovir. Fibrosis data were obtained by liver biopsy or transient elastography.

Exclusion criteria to the study were defined as (i) use of steroids or other immunosuppressive agent in the past 4 weeks; (ii) any disease or clinical test indicating the possibility of a disease or condition that could confound the study results (including, but not limited to: cancer, systemic lupus erythematosus, rheumatoid arthritis or other autoimmune disease); (iii) major surgery or traumatic injury (including blood transfusion) within the past 4 weeks; (iv) use of an investigational drug in the past 12 weeks; (v) HCV, HIV, HDV or HAV coinfection; (vi) significant acute infection such as influenza or other within the past 2 weeks; (vii) history of drug abuse in the past year; (viii) pregnancy or breastfeeding; (ix) patients with: (a) either a medical history or signs of cirrhosis demonstrated by a biopsy result or any other validated non-invasive test revealing cirrhosis, recorded in the patient’s medical file or (b) during the screening visit: a transient elastography value ≥ 10.5 kPa OR a Fibrotest®/Fibrosure® score ≥ 0.48 and an APRI (aspartate aminotransferase platelet ratio Index) score ≥ 1 at screening; history of ascites, gastrointestinal bleeding and/or encephalopathy; any comorbidity likely to lead to liver damage (excessive alcohol consumption; hemochromatosis; Wilson’s disease; autoimmune hepatitis; inflammatory colitis, etc.).

All patients who participated in the study provided prior written informed consent. The study was approved by the French national ethics committee, Comité de Protection des Personnes Ile-de-France.

Blood samples from 10 healthy controls were provided by the Roche medical service. Patients gave informed consent and the blood collection was approved by the Ethikkommission Nordwest- und Zentralschweiz.

### Sample collection and processing

#### Sample collection

Blood samples were collected in Vacutainer Sodium Heparin Tubes. Freshly taken liver biopsies were immediately stored in 5 mL of MACS® Tissue Storage Solution (Miltenyi Biotec). Shipment of samples was temperature controlled with blood samples shipped at room temperature (15–25 °C) and biopsies shipped cooled (2–8 °C). Samples were processed freshly on the same day.

#### PBMC isolation

Isolation of PBMCs from fresh whole blood was performed through density centrifugation. In short, blood was diluted 1:3 with 1X PBS containing 2% heat inactivated FBS. In a 50 mL Leucosep tube (greiner, 227290) 30 mL of diluted blood were layered onto 15 mL of Ficoll-Paque PLUS (GE Healthcare, 17-1440-03) and centrifuged at 800 g for 30′ at room temperature without brake. PBMCs were collected at the interphase, washed with 50 mL isolation buffer and counted for further processing. Freshly isolated PBMCs were either used directly for sorting or frozen in Cryostor CS10 (Stemcell, 07930) for later use.

Generally, all samples used for 10X processing were used freshly. Additionally, a few samples were processed from frozen PBMCs to match exactly the samples used for Smart-seq2 processing and facilitate data integration. Furthermore, in order to increase cell numbers for a few donors (VHB11, VHB34, HC8 and 9) frozen samples were added. The information on fresh vs. frozen processing is available in the metadata of the dataset.

#### Biopsies processing

For preparation of single cell suspensions from biopsy, the biopsy was cut in small pieces with a sterile scalpel and digested in RPMI medium containing 0.25 mg/mL collagenase (Sigma-Aldrich, C6885) and 0.2 mg/mL DNAse (Roche, 1010415900) at 37 °C for 40′ with constant slow shaking. Suspensions were filtered through a 70 μm filter and leftover pieces ground through the filter. The filter was then rinsed with RPMi + 10% FCS. Another filtering step through a 40 μm filter was performed and the filter rinsed with RPMI + 10% FCS.

### Magnetic bead isolation

Magnetic bead isolation was used to isolate CD45^+^ immune cells from fresh biopsy-derived cell suspensions. The cell suspension was first centrifuged at 300 g for 10′. The cell pellet was then washed in 5 mL MACS buffer and pelleted again. CD45^+^ cells were isolated using human CD45 MicroBeads (Miltenyi, 130-045-801) for positive selection according to the manufacturer protocol using MS columns (Miltenyi Biotec, 130-042-201). After magnetic bead isolation cells were pelleted for 10′ at 300 g, supernatant was discarded and cells were resuspended in 40 μL PBS with 0.04% BSA and counted. All biopsy samples were used freshly.

Matched blood samples from biopsy donors were split into two parts with 80 mL used for standard PBMC isolation (see above) and 20 mL used for CD45^+^ magnetic bead isolation to match the isolation procedure of biopsy-derived immune cells. For bead isolation from whole blood StraightFrom Whole Blood CD45 MicroBeads (Miltenyi Biotec, 130-090-872) and the Whole Blood Column Kit (Miltenyi Biotec, 130-093-545) were used according to manufacturer instructions.

### Fluorescence-activated cell sorting (FACS)

For cell staining 1 × 10^6^ fresh or frozen PBMCs were used. First, viability staining was performed using 200 μL of a 1:1000 dilution of the Zombie NIR™ Fixable Viability dye (Biolegend, 423105) for 15′ at room temperature in the dark. Cells were washed with staining buffer (BD Pharmingen) and resuspended in 200 μL Fc-receptor blocking solution, after 5′ the staining mix of antibodies was added and cells were incubated for 20′ at 4 °C in the dark. Cells were then washed twice with CSB and filtered before resuspension to a maximum of 30 × 10^6^/mL for FACS sorting.

For cell staining intended for HBV-specific T cell sorting 2 × 10^6^ PBMCs from HLA-A*02:01^+^ donors were used and stained with 10 μL tetramer (PE-labelled, iTAG MHC Tetramer, FLPSDFFPSV, MBL International Corporation, #TB-0018-1) in 100 μL CSB for 30′ at room temperature in the dark prior to viability and antibody staining described above.

The used antibodies were BUV395-labelled anti-CD4 (BD Biosciences, SK3, #563550), BUV737-labelled anti-CD8 (BD Biosciences, SK1, #564629), BV421-labelled anti-PD-1 (Biolegend, EH12.2H7, #329920), BV510-labelled anti-CD3 (BD Biosciences, UCHT1, #563109), BV711-labelled anti-CD19 (Biolegend, HIB19, #302246), BV711-labelled anti-CD14 (Biolegend, M5E2, #301838), BV711-labelled anti-CD16 (Biolegend, 3G8, #302044), BV711-labelled anti-CD11B (Biolegend, ICRF44, #301344), FITC-labelled anti-CCR7 (Biolegend, G043H7, #353216), BB700-labelled anti-CD45 (BD Biosciences, HI30, #746090), PE-CF594-labelled anti-PD-1, BD Biosciences, EH12.1, #565024), PE-Cy7-labelled, anti-CD45RA (Biolegend, HI100, #304126), APC-labelled anti-CD45RA (Biolegend, HI100, #304112).

FACS sorting was performed on a BD Fusion instrument using BD FACSDiva software version 8.0.1.

### Library preparation and sequencing (10X Genomics)

For scRNA-seq employing the 10X Genomics Chromium platform cell suspensions were used for library preparation following the manufacturer instructions (Chromium Single Cell 3′ Reagent Kits v3 User Guide CG000183 Rev A). A cell recovery of 8000 cells was targeted.

### Smart-seq2

For full-length scRNA-seq of T cells from three HLA-A*02:01^+^ donors, cells were sorted into 96-well plates with one cell per well directly into lysis buffer containing dNTPs and oligo-dT primers. Smart-seq2 library preparation was performed as originally published following the protocol from Picelli *et al*.^37^ using the Nextera XT DNA Library Preparation Kit (96 samples), Illumina, FC-131-1096. For all Smart-seq2 experiments frozen PBMCs were used.

### Sequencing of 10X libraries

10X Libraries were quantified using the Qubit dsDNA HS assay and average library size was calculated running Bioanalyzer DNA High Sensitivity protocol. Libraries were pooled in an equimolar manner and pools were diluted to 2.5 nM before loading into the sequencer. Illumina NovaSeq 6000 instrument was used for sequencing using single-indexed paired-end parameters (28 cycles - 8 cycles - 91 cycles).

### Sequencing of Smart-seq2 libraries

Dual-indexed Smart-seq2 libraries were pooled by equal volumes of library. Each library pool was quantified on a Qubit Fluorometer using the Qubit™ dsDNA HS kit (Thermo Fischer Scientific®). Library quality was assessed on a Bioanalyzer using the Agilent High Sensitivity DNA kit (Agilent Technologies®). Library pools were diluted to 2 nM and sequenced for 2 × 101 cycles on a NovaSeq 6000 instrument (Illumina Inc.).

### Single cell RNA-sequencing data processing

In total, we sequenced 58 experiments on the 10X platform (excluding feature barcoding experiments) and 3 experiments using the Smart-seq2 protocol. We excluded 5 experiments:

One experiment due to inconclusive FACS staining for cell sorting (VHB5_PD1_10X_3p_blood), one experiment because of high mitochondrial gene count, low gene count and low UMI count (VHB17_CD45_10X_3p_blood), three experiments that were re-sequenced because they contained very few cells (VHB34_CD45_10X_3p_blood, VHB27_PD1_10X_3p_blood, and VHB27_NN_10X_3p_blood).

#### 10X data preprocessing

FASTQ files were generated using 10X Genomics cellranger 5.5.0 mkfastq. In order to estimate UMI counts and gene expression levels, reads were mapped to the human genome (hg38) utilizing 10X Genomics cellranger 5.5.0 count. The gene-by-cell count matrix was further processed using Besca^38^ and Scanpy^39^.

#### 10X data filtering

In order to achieve high quality data, only cells that expressed at least 800 and not more than 6.000 genes; included at least 2.500 and not more than 50.000 UMIs; had not more than 15% of UMIs mapping to mitochondrial genes were kept. This resulted in 186,123 total cells.

#### 10X data normalization

Normalization was performed using count depth scaling to 10,000 total counts per cell, resulting in the cp10k (counts per 10,000) unit. Count values were log-transformed using natural logarithm: ln(cp10k + 1).

#### Smart-seq2 experiments

In total, we sequenced 1,713 cells using the Smart-seq2 protocol^37^ from 9 experiments (3 donors × 3 conditions). Two cells were excluded, because the raw sequencing data (FASTQ files) were missing or corrupted (cells 771 and 1320).

#### Smart-seq2 data preprocessing

Base calling was performed with BCL to FASTQ file converter bcl2fastq v2.17.1.14 from Illumina (https://support.illumina.com/downloads.html). In order to estimate gene expression levels, paired-end RNA-Seq reads were mapped to the human genome (hg38) with STAR aligner version 2.5.2a using default mapping parameters^40^. Numbers of mapped reads for all Ensembl transcript variants of a gene (counts) were combined into a single value by featureCounts software^41^ and normalized as TPM (transcripts per million). The gene-by-count matrix was further processed using Besca^38^ and Scanpy^39^.

#### Smart-seq2 data filtering

In order to achieve high quality data, we kept only those cells that expressed at least 800 and not more than 6,000 genes (same as for 10X data); had a percentage of UMIs mapping to mitochondrial genes not more than 15% (same as for 10X data). This resulted in 1,069 total cells.

#### Smart-seq2 data normalization

Normalization was performed using count depth scaling to 10,000 total counts per cell, resulting in the cp10k (counts per 10’000) unit. Count values were log-transformed using natural logarithm: ln(cp10k + 1).

## Data Records

### 10X data

The gene-by-cell raw UMI count matrix and the processed data for the 10X single cell RNA-sequencing experiments are available from Zenodo record 8399409^42^. The raw count matrix is available in the MTX format (barcodes.tsv, genes.tsv, matrix.mtx) together with the corresponding metadata in the TSV format (metadata.tsv) within the gzip archive: raw.tar.gz. The matrix can be processed using Besca^38^. The processed data files are available from the gzip archive standard_workflow_besca2.tar.gz. It contains mainly human-readable text-files or tab-separated files, which can be opened by any text editor or spreadsheet software. The processed data is also available as an AnnData object in the h5ad format: standard_workflow_besca2.h5ad. It can be loaded by Scanpy^39^ for further analyses, by the cellxgene visualization tool^43^, or by other compatible toolkits.

### Smart-seq2 data

The gene-by-cell raw and tpm-normalized count matrices and the processed data for the Smart-seq2 experiments are available from Zenodo record 8399458^44^. The tpm (transcripts per million) normalized count matrix is available in the MTX format (barcodes.tsv, genes.tsv, matrix.mtx) together with the corresponding metadata in the TSV format (metadata.tsv) within the gzip archive: raw.tar.gz. The matrices can be processed using Besca^38^. The processed data files are available from the gzip archive: standard_workflow_besca2.tar.gz. It contains mainly human-readable text-files or tab-separated files, which can be opened by any text editor or spreadsheet software. The processed data is also available as an AnnData object in the h5ad format: standard_workflow_besca2.h5ad. It can be loaded by Scanpy^39^ for further analyses, by the cellxgene visualization tool^43^, or by other compatible toolkits.

### 10X and Smart-seq2 integrated data

The gene-by-cell raw UMI count matrix and the processed data for the integrated data are available from Zenodo record 8399475^45^. The raw count matrix is available in the MTX format (barcodes.tsv, genes.tsv, matrix.mtx) together with the corresponding metadata in the TSV format (metadata.tsv) within the gzip archive: raw.tar.gz. The matrix can be processed using Besca^38^. The processed data files are available from the gzip archive: integrated_10X_SS2.tar.gz. It contains mainly human-readable text-files or tab-separated files, which can be opened by any text editor or spreadsheet software. The processed data is also available as an AnnData object in the h5ad format: integrated_10X_SS2.h5ad. It can be loaded by Scanpy^39^ for further analyses, by the cellxgene visualization tool^43^, or by other compatible toolkits.

## Technical Validation

### Cell sorting

For subsequent scRNA-seq cells were sorted either as bulk populations when used for sequencing with the 10X Genomics platform or directly into lysis buffer of 96-well plates when used for sequencing by the Smart-seq2 protocol. The general gating strategy for these two approaches was identical. Cells were identified as lymphocytes and doublets excluded by forward and side scatter. Viable CD45^+^ cells were selected and CD3^+^ T cells identified.

**Fig. 2:**
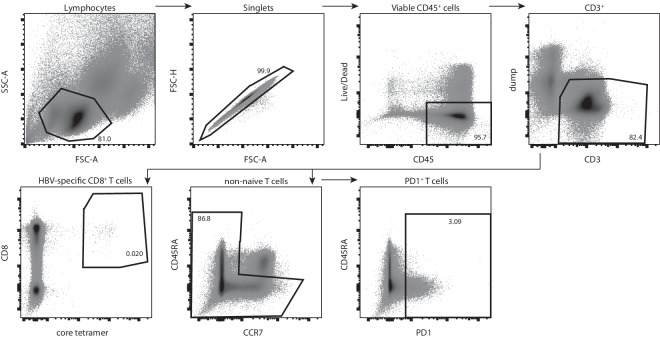
Gating strategy for T cell populations of interest for subsequent scRNA-seq. Representative image of the gating strategy used for cell sorting of non-naive T cells, PD1^+^ T cells and HBV-specific T cells using antibodies against surface markers and fluorescently labeled MHC tetramers against the core protein as indicated on the plot axes.

In the following steps either total non-naive T cells by gating out CD45RA/CCR7 double-positive naive cells, PD1^+^ T cells or HBV specific CD8^+^ T cells were chosen for sorting (Fig. 2). For the sorting of HBV-specific T cells from HLA-A*02:01^+^ donors, cells double-positive for CD8 and the MHC-core-peptide (FLPSDFFPSV) tetramer complex were selected.

**Fig. 3:**
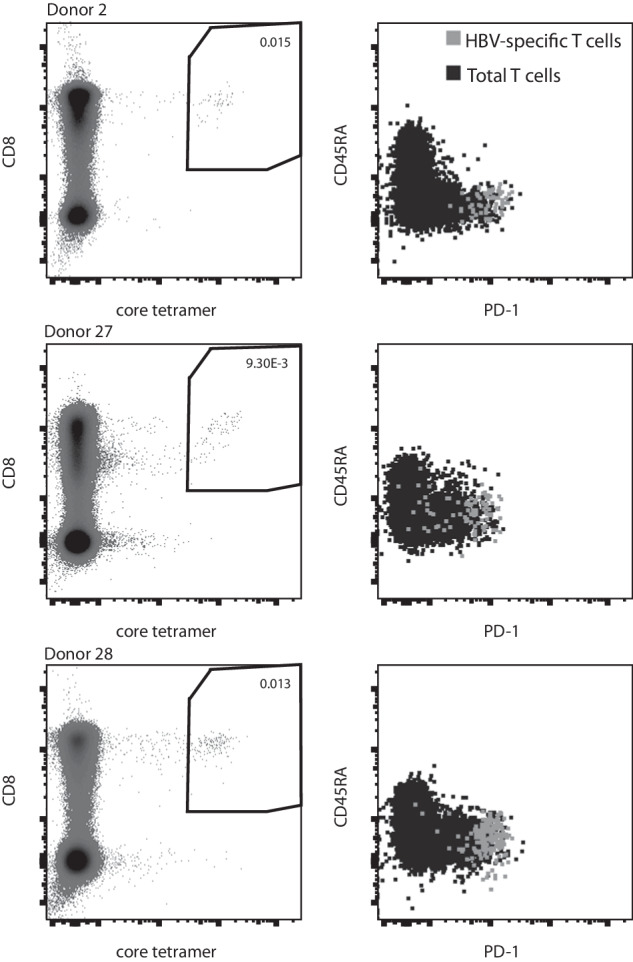
FACS staining and PD1 expression of HBV core-specific CD8 T cells from three donors used for scRNA-seq with Smart-seq2.

For the plate based Smart-seq2 protocol HBV-specific T cells from 3 different donors were included. These cells are assumed to display an exhausted phenotype, indicated by high PD1 expression. Figure 3 shows PD1 expression on HBV-specific cells for each of the donors displayed versus the expression in total T cells. The HBV-specific cell population shows a higher expression of PD1 versus total T cells.

### 10X cell clustering

The steps in this paragraph were done to evaluate the 10X data alone and are not relevant for the integration of the 10X and Smart-seq2 data.

To reduce dataset dimensionality before clustering, the highly variable genes within the dataset were selected. Genes were defined as being highly variable when they have a minimum mean expression of 0.0125, a maximum mean expression of 3 and a minimum dispersion of 0.5.

Technical variance was removed by regressing out the effects of count depth and mitochondrial gene content and the gene expression values were scaled to a mean of 0 and variance of 1 with a maximum value of 10.

**Fig. 4:**
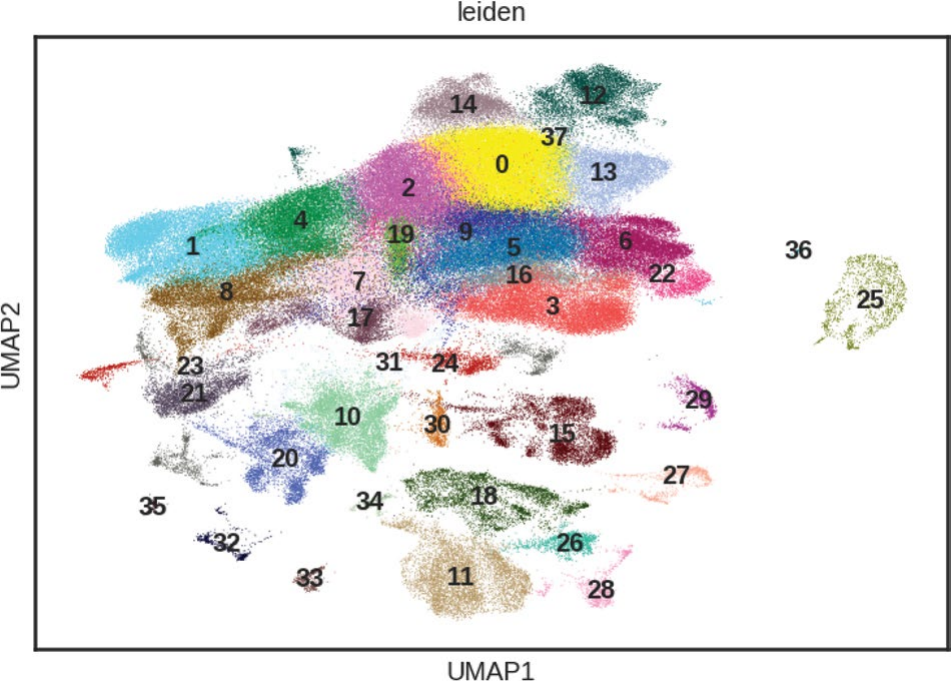
UMAP of 186,123 cells from the 10X platform coloured by 38 Leiden clusters.

The first 50 principal components were calculated and used as input for calculation of the 10 nearest neighbours. The neighbourhood graph was then embedded into two-dimensional space using the Uniform Manifold Approximation and Projection (UMAP) algorithm)^46^. Cell communities are detected using the Leiden algorithm^47^ at a resolution of 1 (Fig. 4).

### Smart-seq2 cell clustering

The steps in this paragraph were done to evaluate the Smart-seq2 data alone and are not relevant for the integration of the 10X and Smart-seq2 data (see paragraph thereafter).

To reduce dataset dimensionality before clustering, the highly variable genes within the dataset were selected. Genes were defined as being highly variable when they have a minimum mean expression of 0.0125, a maximum mean expression of 3 and a minimum dispersion of 0.5.

Technical variance was removed by regressing out the effects of count depth and mitochondrial gene content and the gene expression values were scaled to a mean of 0 and variance of 1 with a maximum value of 10.

**Fig. 5:**
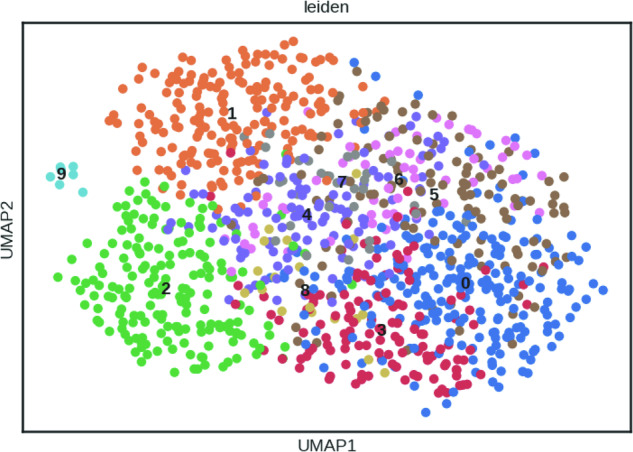
UMAP of 1,069 cells from the Smart-seq2 protocol coloured by 10 Leiden clusters.

The first 50 principal components were calculated and used as input for calculation of the 10 nearest neighbours. The neighbourhood graph was then embedded into two-dimensional space using the UMAP algorithm^46^. Cell communities were detected using the Leiden algorithm^47^ at a resolution of 1 (Fig. 5).

### Integration of 10X and Smart-seq2 data

The raw UMI count matrix (10X data) and TPM count matrix (Smart-seq2 data) were concatenated resulting in 187,192 cells. Normalization was performed using count depth scaling to 10,000 total counts per cell, resulting in the cp10k (counts per 10,000) unit for both protocols. Count values were log-transformed using natural logarithm: ln(cp10k + 1). We did not apply any advanced integration method and therefore cells cluster by protocol (see Fig. 7).

#### Integrated cell clustering

The clustering performed on the integrated dataset is independent from the previous clusterings on the individual datasets (see previous paragraphs).

To reduce dataset dimensionality before clustering, the highly variable genes within the dataset were selected. Genes were defined as being highly variable when they have a minimum mean expression of 0.0125, a maximum mean expression of 3 and a minimum dispersion of 0.5.

Technical variance was removed by regressing out the effects of count depth and mitochondrial gene content and the gene expression values are scaled to a mean of 0 and variance of 1 with a maximum value of 10.

**Fig. 6:**
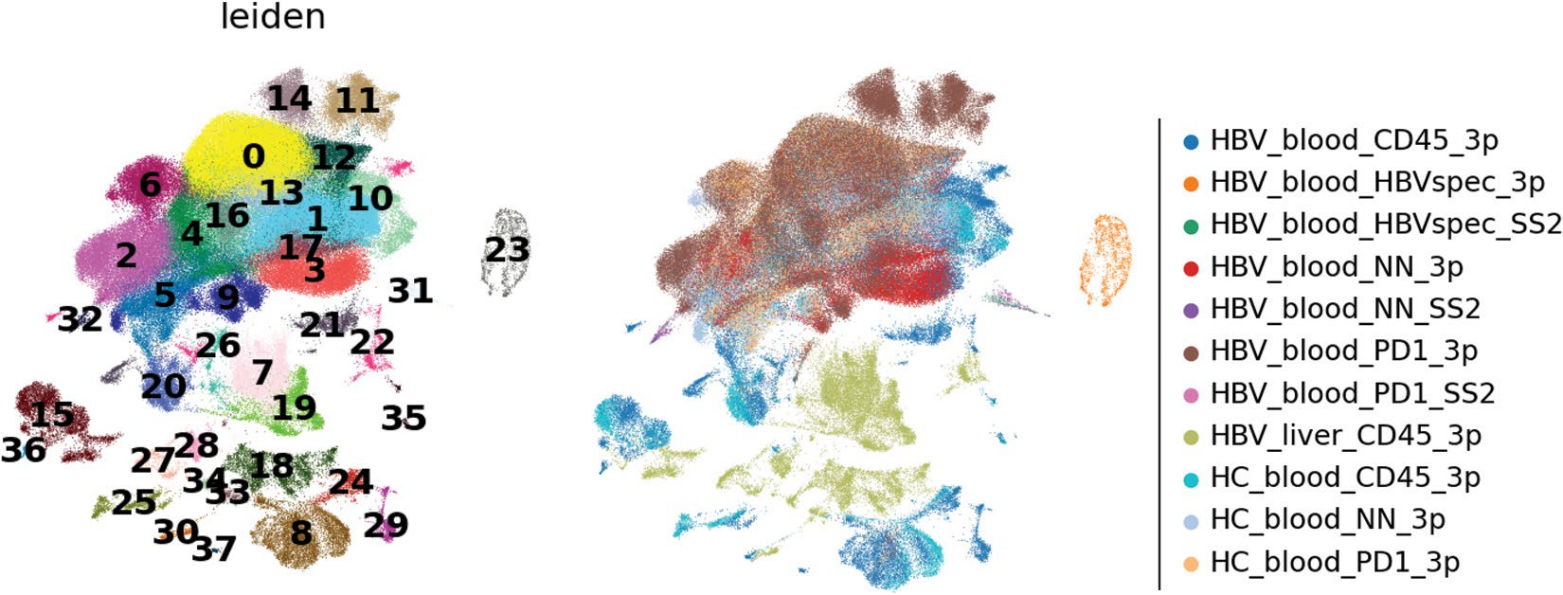
UMAP of 187,192 cells from the integrated 10X and Smart-seq2 dataset coloured by 38 Leiden clusters (left) and experiment condition (right).

**Fig. 7:**
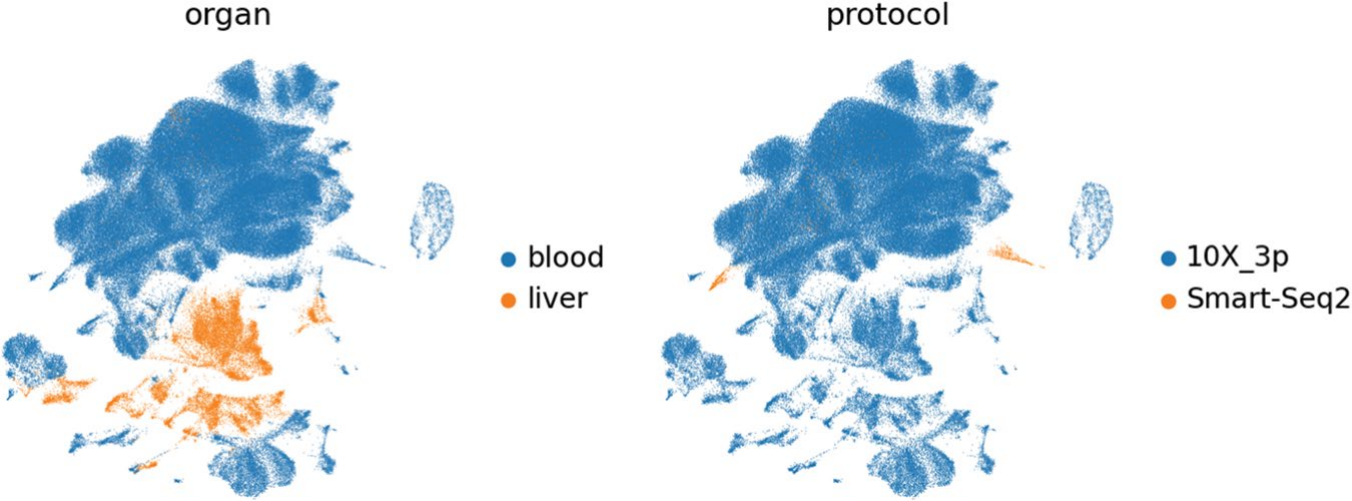
UMAP of 187,192 cells from the integrated 10X and Smart-seq2 dataset coloured by organ (left) and protocol (right).

**Fig. 8:**
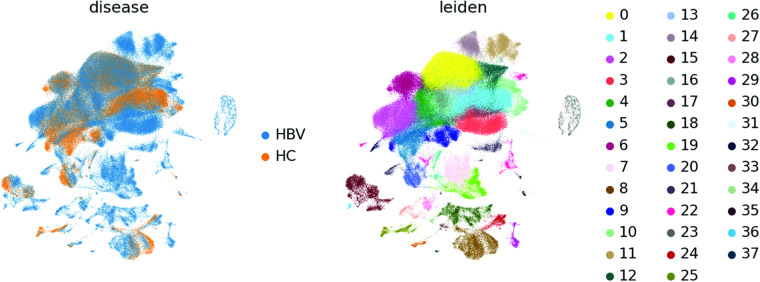
UMAP of 187,192 cells from the integrated 10X and Smart-seq2 dataset coloured by disease (left) and Leiden (right).

The first 50 principal components were calculated and used as input for calculation of the 10 nearest neighbours. The neighbourhood graph was then embedded into two-dimensional space using the UMAP algorithm^46^. Cell communities were detected using the Leiden algorithm^47^ at a resolution of 1 (Figs. 6–8).

We assessed the cell types of all cells by calculating a signature scores for all signatures provided by Besca (https://github.com/bedapub/besca/blob/master/besca/datasets/genesets/CellNames_scseqCMs6_sigs.gmt)^38^. The score is the average expression of a set of genes subtracted with the average expression of a reference set of genes, calculated by Scanpy’s score_genes function (https://scanpy.readthedocs.io/en/stable/generated/scanpy.tl.score_genes.html)^39^.

**Fig. 9:**
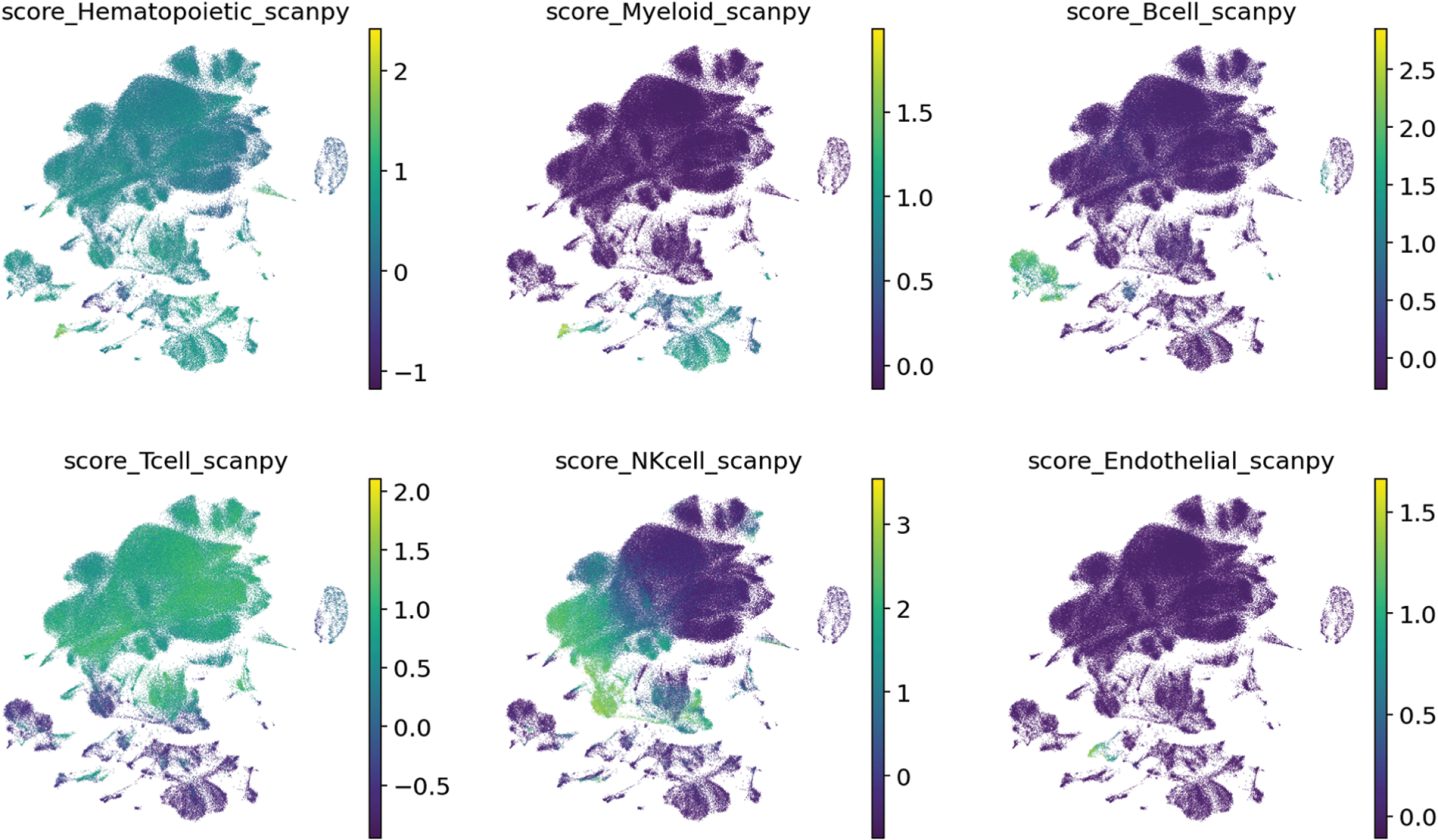
UMAP of 187,192 cells from the integrated 10X and Smart-seq2 dataset coloured by signature score from Scanpy’s score_genes function.

**Fig. 10:**
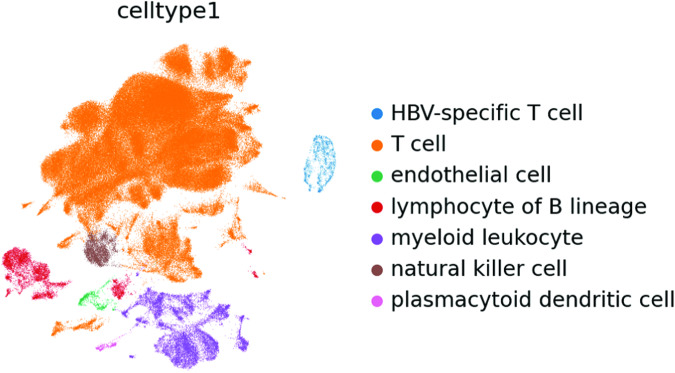
UMAP of 187,192 cells from the integrated 10X and Smart-seq2 dataset coloured by cell type annotation.

These signatures were selected for the cell type annotation (Figs. 9, 10):

- Hematopoietic signature genes: PTPRC, CORO1A, RAC2, CD53, LAPTM5, CXCR4, LCP1
- Myeloid signature genes: CSF3R, MS4A6A, MS4A7, MNDA, C5AR1, FCGR2A, C3AR1, FPR1, LILRB2, HDC, FCGR3B, CCL22
- B cell signature genes: CD19, MS4A1, TNFRSF13C, VPREB3, PAX5, CR2
- T cell signature genes: CD3E, CD3D, CD3G, TRAC, BCL11B, TRAT1, CD2
- NK cell signature genes: NCR1, LIM2, KIR2DL4, KLRC1, IL18RAP, KLRF1,
- Endothelial signature genes: CDH5, ECSCR, CCL14, KDR, TIE1, PCAT19, MYCT1, FLT4

## Data Availability

The Besca^38^ toolkit was used to process the data. The corresponding Jupyter notebooks are available from the Zenodo together with the data. The processing workflow for the 10X data is available from record 8399409^42^ standard_workflow_besca2.ipynb or standard_workflow_besca2.html and the processing workflow for the Smart-seq2 data is available from record 8399458^44^ standard_workflow_besca2.ipynb or standard_workflow_besca2.html. The Jupyter notebook to integrate both datasets is available from record 8399475^45^ integrate_10x_smartseq2.ipynb or integrate_10x_smartseq2.html.
